# Progress toward Understanding the Molecular Basis of Fruit Response to Hypoxia

**DOI:** 10.3390/plants7040078

**Published:** 2018-09-21

**Authors:** Dubravka Cukrov

**Affiliations:** Italian National Research Council (CNR), Via Giuseppe Moruzzi 1, 56127 Pisa, Italy; dubravkacukrov@gmail.com

**Keywords:** oxygen, hypoxia, gas exchange, anaerobic adaptation, N-end rule pathway, ERFs

## Abstract

Oxygen has shaped life on Earth as we know it today. Molecular oxygen is essential for normal cellular function, i.e., plants need oxygen to maintain cellular respiration and for a wide variety of biochemical reactions. When oxygen levels in the cell are lower than levels needed for respiration, then the cell experiences hypoxia. Plants are known to experience root hypoxia during natural environmental conditions like flooding. Fruit, on the other hand, is known to be hypoxic under normal oxygen conditions. This observation could be explained (at least partially) as a consequence of diffusional barriers, low tissue diffusivity, and high oxygen consumption by respiration. From the physiological point of view, hypoxia is known to have a profound impact on fruit development, since it is well documented that a low oxygen environment can significantly delay ripening and senescence of some fruit. This effect of a low-oxygen environment is readily used for optimizing storage conditions and transport, and for prolonging the shelf life of several fruit commodities. Therefore, further understanding of the complex relationship between oxygen availability within the cell and fruit development could assist postharvest management.

## 1. Introduction

Fruit is a reproductive organ of flowering plants (angiosperms), which can be found in a myriad of sizes, shapes, textures, colors, flavors, and odors. Through the course of ripening, the physiology of fruit changes developmentally [1,2], making the ripe fruit attractive to animals, which, by eating the fruit, will disperse the seeds and improve the survival chances of the next generation of the plant [3]. From the human point of view, fruits are a delicious dietary product, highly valued for their nutritive properties [4,5]. On the basis of the ripening behavior, fruit can be classified as climacteric or nonclimacteric [6,7]. In climacteric fruit, ripening is accompanied by a peak in respiration and a concomitant burst of ethylene, whereas in nonclimacteric fruit, respiration shows no dramatic change and ethylene production remains at basal level [8]. As early as 1821, Berard recognized that O_2_ elimination in the storage environment can prevent ripening. Over the course of time, many methods have been developed to efficiently control the ripening of many different fruit commodities. Nowadays, setting the oxygen concentration in storage rooms is one of the most important aspects of postharvest protocols applied to specific fruit, aimed at preserving quality, prolonging shelf life, reducing losses, and obtaining higher market prices [9]. Specifically, controlled atmosphere (CA) storage is based on reducing oxygen and increasing carbon dioxide levels in the environment surrounding the fruit, profoundly affecting general metabolism (respiration in particular), ripening, and senescence [10].

Although oxygen is of paramount importance for normal development, fruits lack an active transport mechanism to distribute oxygen to all cells and a steep oxygen gradient occurs within most fruit tissues, mainly due to respiratory activity [11,12]. Furthermore, different oxygen levels inside the fruit and different tissue diffusivity among varieties could be important factors influencing postharvest quality parameters (such as firmness, shelf life, storage requirements, etc.).

In this paper, first, internal oxygen exchange in the fruit tissue is reviewed. Second, the most important metabolic responses to hypoxia are briefly discussed as well as low-oxygen-based fruit storage. Finally, the oxygen sensing and signaling pathway via N-end rule-mediated degradation of ethylene responsive factor (ERF)-VII proteins is reviewed.

## 2. Oxygen Exchange in Fruit Tissue

Oxygen is one of the most important elements required to sustain life. In order to supply cells with needed oxygen, fruit tissue mainly relays on oxygen transport by diffusion according to Fick’s law, assuming an effective diffusion process, which is driven by concentration gradients [13,14]. However, fruit skin represents a gas diffusion barrier, due to different layers of tissues (aqueous, cuticular, and waxy layers) [15,16,17,18]. Oxygen diffusion through the skin from openings (stomata and lenticels) is proportional to the difference in concentrations of oxygen across the barrier, total area of the skin, solubility of gas in the skin, solid-state diffusion coefficient, and total hole area available on the skin surface (as contributed by openings of stomata and/or lenticels) [18,19]. Therefore, gaseous oxygen first needs to diffuse through the fruit skin, and then, by the process of diffusion, enters into an intercellular system that forms a lattice and serves as the main pathway of oxygen transport [20,21]. However, the efficiency of oxygen transport within the intercellular system will depend on the porosity of the tissue (gas volume/tissue volume), and highly porous tissue has more enhanced oxygen transport compared to less porous tissue [22]. For example, in apple (Red Delicious) the porosity may reach an average of 25% of the total fruit volume [23], while in peach (Miraflores) it accounts for only about 3% of the tissue volume [24] (Table 1).

From the intracellular space, oxygen permeates through the cellular membrane to the cytoplasm, and finally into the mitochondria [21]. When respiration takes place, the concentration gradients appear because of consumption of O_2_ and production of CO_2_ [25,27]. Steep oxygen gradients inside fruit tissues seems to be rather a common phenomenon, especially in large bulky fruit such as pears and apples [28,29]. Oxygen gradients can be observed macroscopically in pears and apples, where the lowest oxygen concentrations are found in the fruit core [27,30]. CO_2_ diffusivity in fruit tissues can be higher than O_2_ diffusivity, causing a larger outflow of CO_2_ than inflow of O_2_, and a pressure difference between the inside of the fruit and the external atmosphere may develop. This is probably due to the greater solubility of CO_2_ than O_2_ in water [27]. Hence, besides gas diffusion driven by concentration gradients, gas exchange in the fruit may occur by permeation due to pressure gradients in the fruit tissues [27,31]. Additionally, when observed microscopically, much more gas diffuses through the pore and cell wall network than through the cytoplasm; therefore, cytoplasm has much lower oxygen concentration than intracellular space [21]. Therefore, hypoxia can occur under normal air conditions in some fruit (especially in the core tissue). However, it is important to note that oxygen gradient can affect fruit physiology profoundly, since hypoxia is known to affect several different biochemical processes.

## 3. Effects of Oxygen Availability on Metabolism and Development

Hypoxia is a condition in which a deficiency of cellular oxygen limits mitochondrial respiration, whereas anoxia is a condition in which the fluxes of respiratory gases are restricted and adenosine triphosphate (ATP) produced by mitochondria is negligible relative to ATP produced by glycolysis [32], which eventually will lead to cell death [33]. However, as mentioned previously, fruit tissue (especially the cortex of bulky fruit) can be hypoxic at normal air oxygen concentrations [12,27]. Respiration by the fruit tissues and barriers of diffusion/exchange of gases as posed by anatomical, morphological, physical, and/or biochemical components (present either on the surface or inside the fruit) are the factors responsible for the gradual lowering of the O_2_-to-CO_2_ ratio from outside to inside the fruit [34]. This tissue-specific hypoxia under normal air oxygen concentrations was observed also in other plant organs (roots, rhizomes, seeds, coleoptiles) and was shown to lead to the development and growth of an anaerobic core in roots [35,36].

Fruit response to hypoxia is mainly reflected in a reduction of cellular respiration followed by induction of anaerobic respiration, which leads to the fast breakdown of sugars [37,38]. Low oxygen is known to affect the transport chain [39,40], glycolysis [41], ethanolic fermentation [42], tricarboxylic acid (TCA) cycle [43], Yang cycle [44], and amino acid metabolism [45]. Recently, cell death was correlated with steep O_2_ gradients across the skin and toward the middle of the mesocarp in grape berries [46]. 

Several comprehensive transcriptomic studies of hypoxic fruit tissue resulted in the identification of the whole set of differentially expressed genes, providing a descriptive overview of the fruit-specific transcriptomic adjustments to oxygen deprivation [47,48,49]. For example, gene expression of alcohol dehydrogenase (ADH) is correlated with oxygen availability [50], and enzymatic activity of ADH and pyruvate decarboxylase (PDC) increases under hypoxia [39,51,52].

Hypoxia also decreases ethylene production and tissue sensitivity to ethylene [53]. Specifically, oxygen is a substrate for the reaction catalyzed by 1-aminocyclopropane-1-carboxylic acid (ACC) ACC-oxidase, an enzyme involved in ethylene biosynthesis. Therefore, oxygen is required for the synthesis as well as the action of ethylene in fruits [13,51,54]. Another important effect of hypoxia on fruit physiology is delayed ripening and senescence. This effect of hypoxia is adapted in agricultural storage methods, therefore it comes as no surprise that most of our understanding of fruit response to hypoxia comes from the research of fruit response to low-oxygen storage.

### Physiological Effects of Low-Oxygen-Based Storage

As mentioned previously, one of the most important effects of hypoxia is delayed ripening and senescence of some fruit, especially considering its application in commercial fruit storage, transport, and packaging. This effect of hypoxia is adapted in the so-called controlled atmosphere (CA) storage method, in which the concentrations of oxygen, carbon dioxide, and nitrogen are strictly controlled along with the temperature and humidity of the storage room [10]. On the other hand, modified atmosphere (MA) is the practice of modifying the composition of the internal atmosphere of a package of fruit (fresh or cut) in order to improve the shelf life [9].

The most common CA method is used on apples and pears, in which the combination of altered atmospheric conditions and reduced temperature allows prolonged storage. Storage can reach up to one year for some apple varieties (Granny Smith) when applying a CA-based storage protocol [10]. Apart from delaying ripening, CA-based storage has been shown to have several other important positive effects in terms of postharvest parameters. In apple fruit, for example, CA storage is known to enhance several desirable attributes, such as firmness and taste retention, higher titratable acidity, maintenance of soluble solid content, and better skin color [9,10]. Furthermore, low-oxygen storage may also be effective in controlling superficial scald, a symptom of chilling injury that commonly occurs, for example, in Granny Smith apples [55]. However, if not carefully managed, CA storage can result in negative organoleptic/sensorial parameters, such as the production of off-flavors due to ethanol and acetaldehyde accumulation [9,10], underlining the importance of understanding the physiologic response to low oxygen in the storage room.

Physiologically, a low-oxygen storage effect on climacteric fruit ripening is reflected through O_2_ influence on respiration, largely through its inhibitory effect on ethylene action [56]. On the molecular level, the action of low oxygen on fruit ripening involves suppression of the de novo synthesized ripening proteins and mRNA transcripts, induction of new proteins and mRNA species, and constitutive expression of preexisting proteins or mRNA species [50,51]. Furthermore, hypoxia appears to modulate not only gene expression but also posttranscriptional and posttranslational processes [47]. Additionally, low-oxygen storage is known to affect production of volatiles, cell wall–related metabolism, and overall energy-related metabolism [51,57,58], and it can reduce cytosolic pH and energy charge [39,40].

These studies indicate that low-oxygen-based storage affects ripening at different metabolic and molecular levels, pointing out the importance of such research efforts. Additionally, understanding metabolomic changes and identifying metabolic markers during low-oxygen-storage could be useful in optimizing protocols. 

## 4. Activation of the Anaerobic Response by the N-End Rule Pathway

In several plant species (*Arabidopsis*, rice, and poplar), a set of 49 genes were identified as ubiquitously induced upon hypoxia and/or submergence, therefore, were named core hypoxia-responsive genes (HRGs) [59,60,61]. HRGs are encoding enzymes involved in sucrose catabolism, anaerobic fermentation (alcohol dehydrogenase and pyruvate decarboxylase), reactive oxygen species regulation, gene transcription, and proteins of unknown function [60]. Major transcriptional control over HRGs is exerted by Group VII ethylene response factors (ERFs), as these transcriptional factors are known to upregulate around 50% of *A. thaliana* HRGs [62,63,64]. Furthermore, an evolutionarily conserved 12 bp cis-regulatory motif, named hypoxia-responsive promoter element (HRPE), is enriched in promoters of hypoxia-responsive genes in multiple species. In *Arabidopsis*, 39 of the 49 core HRGs were identified as putative direct targets of the ERF-VIIs that are stabilized in oxygen-deficient cells, based on detectable HRPE [65].

*A. thaliana* encodes five ERF-VIIs (Table 2), two of which, HYPOXIA RESPONSIVE ERF1 (HRE1) and HRE2, are HRGs [60,65,66], whereas ERF-VII transcripts RELATED TO APETALA2.12 (RAP2.12), RAP2.2, and RAP2.3 accumulate constitutively under normoxic conditions [65,66,67,68,69]. RAP 2.3 and RAP 2.12 can bind to acyl-CoA-binding protein (ACBP) [63,70,71], which prevents their movement into the nucleus under aerobic conditions and protects them against proteasomal degradation in air [63]. Upon hypoxia, ERF-VII transcriptional factors (TFs) move into the nucleus, where they activate anaerobic gene expression. RAP2.2 and RAP2.12 are necessary to activate the transcription of anaerobic genes, whereas HREs sustain their transcription after activation by RAPs [63,65,72]. Upon reoxygenation, RAP2.12 is rapidly degraded via the N-end rule pathway and proteasome-mediated proteolysis to downregulate the hypoxic response [62,63].

In *Arabidopsis*, oxygen-dependent modification and targeted proteolysis of ERF-VII TFs is determined by their conserved N-terminal motif (Met-Cys-Gly-Gly-Ala-Ile/Leu, MCGGAI/L, termed the MC motif), which is recognized by the enzymes involved in the N-end rule pathway [63]. First, the terminal Met is removed from the protein by methionine aminopeptidase (MAP1/2), leaving the second amino acid of the ERF-VII protein (Cys) exposed [73]. This residue is then oxidized to cysteine sulphenic acid in an oxygen-dependent manner catalyzed by plant cysteine oxidase (PCO1/2) [74], followed by transfer of the Arg of amino-acylated tRNAArg to the N terminus of the oxidized NH2-Cys-ERF-VII, catalyzed by arginyl-tRNA transferase (ATE1/2). This triggers subsequent ubiquitination by the single subunit E3 ubiquitin ligase proteolysis 6 (PRT6) and targets the protein to the proteasome for degradation, which can occur in both the cytosol and the nucleus [62,75].

### Oxygen-Dependent Degradation of Fruit-Specific ERF VII

The existence of the same mechanism of anaerobic response activation by the N-end rule pathway was demonstrated in apple fruit during low-oxygen storage [47] and partially in transgenic tomato fruit [76].

**Figure 1:**
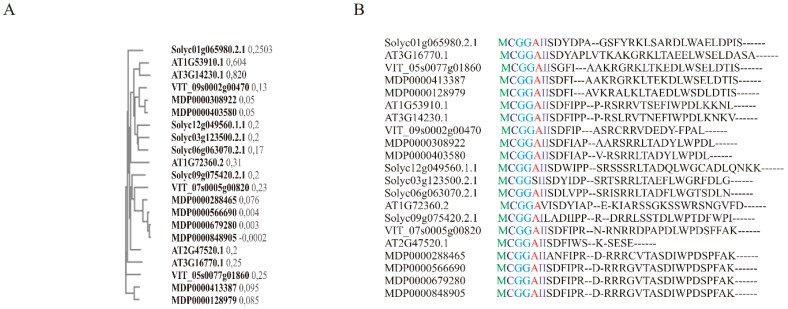
ERF-VII protein sequence similarity among *Arabidopsis*, tomato, grapevine, and apple fruit. (**A**) Between-species phylogenetic tree representing relationships among ERF-VII proteins (represented values are real distance). (**B**) Multiple sequence alignment of ERF-VII proteins. Conserved MCGGAI amino acid sequence can clearly be seen. This amino acid sequence is recognized by the enzymes involved in the N-end rule pathway. Multiple sequence alignments were performed using Clustal Omega software (EMBL-EBI, September 2018).

**Figure 2:**
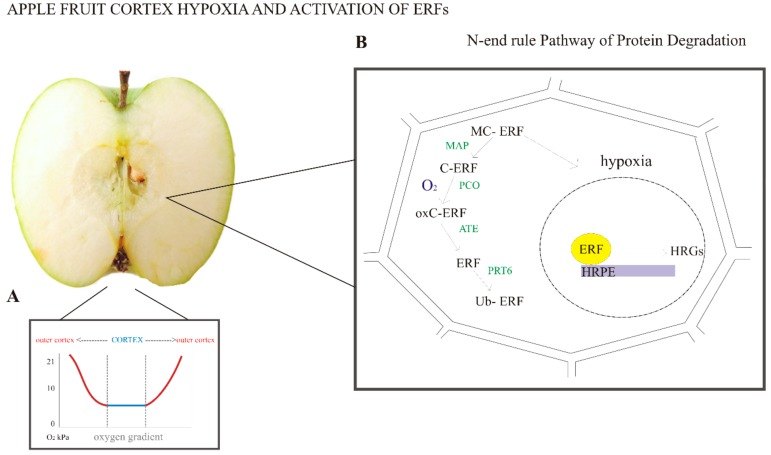
Fruit tissue hypoxia and oxygen signaling pathway. (**A**) Bulky fruit, such as apple, can be hypoxic under a normal oxygen environment, where the lowest oxygen concentrations are found in the cortex. The graph illustrates the possible gradient formed inside the fruit in normal oxygen conditions (adapted from Paul and Pandey [34]). (**B**) The oxygen concentration in the cortex can reach very low values, and the cell can experience hypoxia. Hypoxia triggers the accumulation of ERFs in the nucleus, which can bind the hypoxia-responsive promoter element (HRPE) sequence, which in turn activates transcription of hypoxia-responsive genes (HRGs). On the other hand, under normal oxygen conditions, ERFs are subjected to enzymatic degradation via the N-end rule pathway. Enzymes are shown in green. MAP, methionine aminopeptidase; PCO, plant cysteine oxidase; ATE, arginyl-tRNA transferase; PRT6-E3, ubiquitin ligase proteolysis; MC-ERF, amino acid sequence of the protein; oxC-ERF, oxidation of the cysteine residue; Ub-ERF, ubiquitination of ERF. Although available literature indicates ERF degradation via the N-end rule pathway, genes coding the enzymes involved in the N-end rule pathway of protein degradation have yet to be identified. Therefore, this is an illustration of ERF oxygen signaling based on knowledge obtained in Arabidopsis and apple fruit (adapted from Licausi et al. [64]).

The apple genome contains eight genes coding for ERF-VII TFs [77], distinguished by a conserved N-terminal consensus MCGGAI (Figure 1). Putative apple ERF proteins (orthologous to the oxygen sensors AtRAP2.12 and AtRAP2.2) were found to be stabilized under hypoxic conditions (Figure 2), suggesting that the oxygen-sensing mechanism based on the N-end rule pathway and on the posttranslational regulation of group VII ERF protein is also present and active in apple fruit [47].

In tomato, there are five members of ERF subgroup E (AtERF VII orthologous genes), expressed in reproductive tissues (E1 and E2), vegetative tissue (E3), or ubiquitously (E4) [78]. Furthermore, these TFs are regulated by ethylene, and in the case of E1 also by auxin [78]. Lee et al. (2012) demonstrated that ERF6 (ERF.E4) acts as a negative regulator of carotenoid accumulation during tomato fruit ripening [79]. Recently, Liu et al. (2016) demonstrated that expression of ERF.E1, ERF.E2, and ERF.E4 is suppressed in the tomato-ripening mutants, suggesting their important regulatory role in fruit ripening [80]. Additionally, it seems that ERF.E members are the most active ERFs in ethylene- and RIN/NOR-dependent ripening [80]. All members of this tomato protein subgroup are characterized by the presence of an MCGGAI motif at the N-terminus, which qualifies ERF.E proteins as candidate substrates of the N-end rule pathway (Figure 1 and Figure 2). Research conducted on tomato suggested that ERF E genes are involved not only in the activation of anaerobic response by the N-end rule pathway, but also in fruit ripening and development, such as regulation of carotenoids [47,79], indicating the importance of understanding the relationship between hypoxia signaling via ERFs and fruit ripening. 

Recently, Meitha et al. identified three ERF-VIIs in grape berries, which are substrates for oxygen-dependent N-end rule proteolysis as in *Arabidopsis* [81]. Additionally, several hypoxia-responsive ERFs were shown to be directly involved in transcriptional regulation of anaerobic metabolism genes involved in persimmon deastringency [82,83,84]; however, their possible oxygen-dependent regulation via the N-end rule pathway is still unknown. Although it is worth noting that DkERF10, based on an amino acid sequence, could be a candidate substrate of the N-end rule pathway of protein degradation. A recent study by Zhu et al. (2018) demonstrated the existence of a transcriptional cascade involving ERF-VII (DkERF9, 10, 18, 19, 21, 22) and MYB transcriptional factors (DkMYB6 and 10) that led to upregulation of the fermentation genes (DkADH1 and DkPDC2) [85].

Taken together, the results obtained for fruit-specific ERF genes (orthologous Arabidopsis ERF-VII) contribute to further understanding of the molecular basis of fruit response to hypoxia. Additionally, ERF-VII’s role in fruit hypoxia response and its physiological role during ripening highlights the importance of future research aimed at further characterization of its role in fruit and the complex oxygen-dependent regulatory mechanism. 

## 5. Conclusions

In the last decade, increasing efforts of the scientific community have contributed greatly to our understanding of fruit low-oxygen response. Understanding the O_2_ response of fruit requires knowledge of fruit macrostructure, gas-exchange properties of the tissues, cellular microstructure, and respiration kinetics. To date, the complexity of fruit tissue response to hypoxia has been well explained, especially for bulky fruit during low-oxygen storage. Furthermore, the list of newly identified compounds (metabolites, volatiles, and specific proteins) is growing daily; this not only extends our understanding of complex metabolic response to low oxygen but also contributes greatly to fruit storage and transport optimization. Transcriptional control of around 50% of low-oxygen-responsive genes is regulated by ERFs (subgroup VII). Considering the important role of these ERFs in oxygen-sensing mechanisms, and the fact that fruit tissue is hypoxic under normal oxygen concentrations, it would be interesting to further investigate the possible involvement of ERFs in fruit ripening and development. An understanding of the changes in the metabolome, gene transcription patterns, and regulatory mechanisms and their complex relationship not only contributes to our understanding of molecular responses but can also be used to maximize fruit quality after harvest and during storage and could assist in fruit breeding.

## Figures and Tables

**Table 1:** Porosity of some climacteric and nonclimacteric raw fruits [24,25,26].

Fruit	Porosity (%)	Respiration
Apple (Granny Smith)	23.8	Climacteric
Strawberry (Chandler)	6.3	Nonclimacteric
Peach (Miraflores)	2.6	Climacteric
Mango (Tommy Atkins)	9.9	Climacteric
Nectarine (Sunglo)	4.0	Climacteric
Orange (Thomson)	48.5	Nonclimacteric
Pear (Hosui)	1.7	Climacteric

**Table 2:** Gene nomenclature of ethylene response factor (ERF) subgroup VII proteins in *Arabidopsis*, tomato, apple, and grapevine. *Arabidopsis* and tomato have five genes in the ERF-VII subfamily, apple has eight, and grapevine has three.

Species	Gene ID	Symbol
*Solanum lycopersicum*	Solyc09g075420	*ERF2b* (*ERF.E.1*) *
	Solyc06g063070	*JERF1* (*ERF.E.2*)
	Solyc03g123500	*JERF3* (*ERF.E.3*)
	Solyc01g65980	*ERF 6* (*ERF.E.4*)
	Soyc12g049560	*ERF3* (*ERF.E.5*)
*Arabidopsis thaliana*	AT1G53910.1	*RAP2.12*
	AT1G72360.2	*HRE1*
	AT2G47520.1	*HRE2*
	AT3G14230.1	*RAP2.2*
	AT3G16770.1	*RAP2.3*
*Malus x domestica*	MDP0000308922	*Mdo006699*
	MDP0000403580	*Mdo006712*
	MDP0000679280	*-*
	MDP0000566690	*-*
	MDP0000413387	*Mdo002990*
	MDP0000128979	*Mdo003328*
	MDP0000848905	*Mdo002404*
	MDP0000288465	*Mdo002401*
*Vitis vinifera*	VIT_07s0005g00820	*VvERF057*
	VIT_05s077g01860	*VvERF058*
	VIT_09s0002g00470	*VvERF059*

* Gene nomenclature proposed by Pirrello et al. (2012) [74].
